# Complete Genome Sequence and Comparative Analysis of the Fish Pathogen *Lactococcus garvieae*

**DOI:** 10.1371/journal.pone.0023184

**Published:** 2011-08-04

**Authors:** Hidetoshi Morita, Hidehiro Toh, Kenshiro Oshima, Mariko Yoshizaki, Michiko Kawanishi, Kohei Nakaya, Takehito Suzuki, Eiji Miyauchi, Yasuo Ishii, Soichi Tanabe, Masaru Murakami, Masahira Hattori

**Affiliations:** 1 School of Veterinary Medicine, Azabu University, Sagamihara, Kanagawa, Japan; 2 Planning Office for the Center for Computational and Quantitative Life Science, RIKEN, Yokohama, Kanagawa, Japan; 3 Graduate School of Frontier Sciences, University of Tokyo, Kashiwa, Chiba, Japan; 4 National Veterinary Assay Laboratory, Ministry of Agriculture, Forestry and Fisheries, Kokubunji, Tokyo, Japan; 5 Graduate School of Biosphere Science, Hiroshima University, Higashi-Hiroshima, Hiroshima, Japan; J. Craig Venter Institute, United States of America

## Abstract

*Lactococcus garvieae* causes fatal haemorrhagic septicaemia in fish such as yellowtail. The comparative analysis of genomes of a virulent strain Lg2 and a non-virulent strain ATCC 49156 of *L. garvieae* revealed that the two strains shared a high degree of sequence identity, but Lg2 had a 16.5-kb capsule gene cluster that is absent in ATCC 49156. The capsule gene cluster was composed of 15 genes, of which eight genes are highly conserved with those in exopolysaccharide biosynthesis gene cluster often found in *Lactococcus lactis* strains. Sequence analysis of the capsule gene cluster in the less virulent strain *L. garvieae* Lg2-S, Lg2-derived strain, showed that two conserved genes were disrupted by a single base pair deletion, respectively. These results strongly suggest that the capsule is crucial for virulence of Lg2. The capsule gene cluster of Lg2 may be a genomic island from several features such as the presence of insertion sequences flanked on both ends, different GC content from the chromosomal average, integration into the locus syntenic to other lactococcal genome sequences, and distribution in human gut microbiomes. The analysis also predicted other potential virulence factors such as haemolysin. The present study provides new insights into understanding of the virulence mechanisms of *L. garvieae* in fish.

## Introduction

*Lactococcus garvieae* (formerly *Enterococcus seriolicida*) is a major pathogen of fish and causes fatal haemorrhagic septicaemia in fish such as yellowtail and trout. The septicaemic infection produced by *L. garvieae* is termed lactococcosis to distinguish it from streptococcosis, and causes important economic losses in the fish farming industry [1]. *L. garvieae* has also been isolated from buffaloes with mastitis [2], and clinical specimens of human blood, urine, and skin [3]–[5]. For this reason, *L. garvieae* is considered an emerging zoonotic pathogen. However, the pathogenic mechanisms of this bacterium are poorly understood. It has only been demonstrated that virulence of *L. garvieae* for fish is, in part, dependent on its ability to form a capsule [6]–[8]. *L. garvieae* isolated from diseased fish is classified serologically into two groups, KG− and KG+, and capsulated *L. garvieae* (KG−) are more virulent in fish than non-capsulated *L. garvieae* (KG+) [7]–[9].

In the genus *Lactococcus*, *Lactococcus lactis* is the most well studied *Lactococcus* species. *L. lactis* used in dairy fermentations are generally regarded as safe (GRAS) for human consumption [10]. *L. garvieae* and *L. lactis* are closely related phenotypically, and there is currently no simple means of distinguishing between these taxa [1]. The genomes of five *L. lactis* strains (IL1403, KF147, MG1363, NZ9000, and SK11) have been fully sequenced to date [11]–[15]. In contrast, the genomic characteristics of *L. garvieae* have not been described.

Comparison of the genome sequences of pathogenic and non-pathogenic strains can provide useful information on genes that are specific for a virulent isolate. We determined the complete genome sequences of a virulent strain Lg2 (serotype KG−) and a non-virulent strain ATCC 49156 (serotype KG+) of the fish pathogen, *L. garvieae*. *L. garvieae* ATCC 49156 was originally isolated in 1974 from diseased yellowtail as *E. seriolicida* ATCC 49156^T^ [16], has undergone phenotypic changes during its descent from the ancestral strain, and now is non-pathogenic to yellowtails [17]. *L. garvieae* Lg2, isolated in 2002 from diseased yellowtail, is highly virulent and α-haemolytic [17]. From the present study including the whole genome analysis of the Lg2 and the ATCC 49156 genomes and their comparative analysis with other related genomes as well as the analysis of the capsule gene cluster in the less virulent strain Lg2-S, we strongly suggest that the capsule is crucial for pathogenesis of Lg2.

## Materials and Methods

### *Lactococcus garvieae* strains

*L. garvieae* ATCC 49156 was obtained from the American Type Culture Collection (ATCC). This strain was isolated in 1974 from diseased yellowtail in Japan as a fish pathogen, *E. seriolicida* ATCC 49156^T^ [16]; *E. seriolicida* was reclassified as *L. garvieae* [2]. The isolate was subcultured in EF agar supplemented with 2,3,5-triphenyltetrazolium chloride (TTC) many times in the ATCC, and now is non-pathogenic to yellowtails [17]. *L. garvieae* Lg2 was isolated in 2002 from yellowtail that showed signs of lactococcosis in Japan [17]. The degree of virulence was calculated [50% lethal dose (LD_50_)] and the LD_50_ value of Lg2 is <10^2^ colony-forming unit (CFU) per fish. The mutant Lg2-S was derived from Lg2 after subculturing on Todd-Hewitt agar (Becton, Dickinson and Company) supplemented with TTC 4 times; the LD_50_ value of Lg2-S is >10^8^ CFU per fish [8]. These strains were cultured in Todd-Hewitt agar for 20 h at 25°C, suspended in 20% glycerol solution, and stored at −80°C.

### Genome Sequencing

The genome sequences of ATCC 49156 and Lg2 were determined by a whole-genome shotgun strategy. We constructed genomic libraries containing 2-kb inserts, and generated 39,283 sequences (9.6-fold coverage) for ATCC 49156 and 30,720 sequences (10.6-fold coverage) for Lg2 from both ends of the genomic clones using ABI 3730xl sequencers (Applied Biosystems). Sequence reads were assembled with the Phred-Phrap-Consed program [18] and gaps were closed by direct sequencing of clones that spanned the gaps or of PCR products amplified with oligonucleotide primers designed to anneal to each end of neighbouring contigs. The overall accuracy of the finished sequence was estimated to have an error rate of <1 per 10,000 bases (Phrap score of ≥40).

### Informatics

An initial set of predicted protein-coding genes was identified using Glimmer 2.0 [19]. Genes consisting of <120 bp and those containing overlaps were eliminated. All predicted proteins were searched against a non-redundant protein database (nr, NCBI) using BLASTP with a bit-score cut-off of 60. The start codon of each protein-coding gene was manually refined from BLASTP alignments. The tRNA genes were predicted by the tRNAscan-SE [20] and the rRNA genes were detected by BLASTN using known *Lactococcus* rRNA sequences as queries. Protein domains were identified using the Pfam database. Orthology across the whole genomes was determined using BLASTP reciprocal best hits with a bit-score cutoff of 60 in all-against-all comparisons of amino acid sequences. These sequences were concatenated and aligned by the neighbour-joining method with 1,000 bootstrap iterations using ClustalW. The genome sequence data of *L. garvieae* ATCC 49156 and Lg2 have been deposited in DDBJ/GenBank/EMBL under accession numbers AP009332 (ATCC 49156) and AP009333 (Lg2). The sequences of the *epsD* and *epsL* genes of *L. garvieae* Lg2-S are available under accession numbers AB576165 and AB576166, respectively.

### Reverse transcriptase-polymerase chain reaction (RT-PCR)

For evaluation of mRNA expression, total RNA from *L. garvieae* Lg2 and Lg2-S was extracted using TRIzol reagent (Invitrogen). RNA was quantified by spectrophotometry. RNA (40 pg/µL) was subjected to semi-quantitative RT-PCR with SuperScriptTM One-Step RT-PCR kit with PlatinumTM Taq (Invitrogen) and a TP-3000 thermal cycler (TaKaRa Bio). Primer sequences are listed in Table S4. Oligonucleotide primers were purchased from Greiner (Japan). RT was carried out at 50°C for 30 min. The PCR cycling conditions were as follows: 94°C for 2 min, followed by 40 cycles of 94°C for 15 s, 56°C for 30 s, and 72°C for 1 min. The annealing temperatures and amplification cycles were determined by preliminary experiments to obtain semi-quantitative results. After amplification, PCR products were electrophoresed on 2% agarose gel (Agarose X, NIPPON GENE), stained with ethidium bromide, and photographed under ultraviolet illumination. They were then compared with a known standard [Hyper Ladder IV (100 bp), Bioline] for size determination.

### PCR of the capsule gene cluster in human faecal bacteria

Faecal samples were obtained from healthy human individuals. All subjects gave written informed consent for their participation. The study was performed in accordance with the Declaration of Helsinki, and the Human Research Ethics Committee of Azabu University approved the study (approval number 0721). The purification of bacterial genomic DNA from the faeces was carried out as described previously [21]. Purified achromopeptidase (Wako, final conc. 2,000 units/mL of cell suspension) was used. The sequences of the capsule gene clusters of *L. garvieae* Lg2 and *L. lactis* subsp. *lactis* KF147 were aligned and the sets of consensus primers, 1823F (5′-TCGCTGTTGCTTCTATAGCCTAC-3′) and 70R (5′-GGTCGTGACTAAACAACAATTCTG-3′); 958F (5′-GTACGGCTCGATCATCTTGAC-3′) and 3321R (5′-TATTGGACAACACATGGTCGAAG-3′); 3262F (5′-GAGTTCTAAATCTGCTCGTTGAGG-3′) and 5942R (5′-TTAACCAAGTTATTGTTAGTCCAGTC-3′); 5138F (5′-CGGTTTACGAAGATCACCATC-3′) and 6736R (5′-TTGGCAATCTGATTCAGTTATTTC-3′); 5524F (5′-AGTTGTTACACCAAGAACCGTGAG-3′) and 8270R (5′-TCCTCGGTCATTGAACTATCAG-3′) were created. The genes in the capsule gene cluster were amplified from the faecal DNAs using these PCR primer sets. PCR was performed using a T1 Thermo cycler (Biometra) with 40 cycles of denaturation (30 sec at 96°C), annealing (20 sec at 58°C), and elongation (5 min at 68°C), with a final extension at 72°C for 10 min. Amplified products were verified by electrophoresis on 0.8% agarose gel and sequenced.

## Results and Discussion

### General features of the genomes of *L. garvieae* and comparative analysis

**Figure 1:**
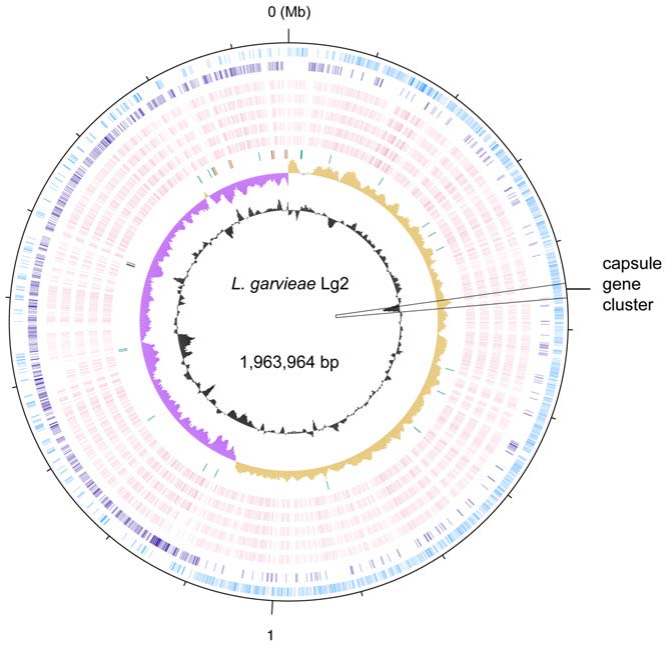
Circular representation of the chromosome of *L. garvieae* Lg2. From the outside in: circles 1 and 2 of the chromosome show the positions of protein-coding genes on the positive and negative strands, respectively. Circles 3–6 show the positions of protein-coding genes that have orthologs in *L. lactis* subsp. *lactis* IL1403, *L. lactis* subsp. *lactis* KF147, *L. lactis* subsp. *cremoris* MG1363, *L. lactis* subsp. *cremoris* SK11, and *L. lactis* subsp. *cremoris* NZ9000. Circle 7 shows the positions of tRNA genes (purple) and rRNA genes (brown). Circle 8 shows a plot of GC skew [(G−C)/(G+C); khaki indicates values>0; purple indicates values<0]. Circle 9 shows a plot of G+C content (outward: higher values than the average).

**Figure 2:**
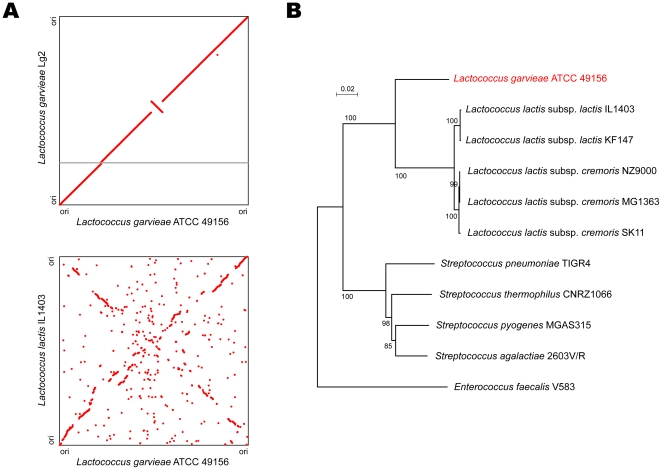
Genome-based phylogenetic analysis of *L. garvieae*. (**A**) Synteny between the *L. garvieae* and *L. lactis* subsp. *lactis* IL1403 chromosomes. Each plot point represents reciprocal best matches by BLASTP comparisons between orthologs. Genome numbering initiated at *dnaA* for both chromosomes. Shaded region indicates the location of the capsule gene cluster in the Lg2 genome. (**B**) Phylogenetic relationships between the genomes of sequenced *Streptococcaceae* inferred from 27 concatenated ribosomal protein amino acid sequences. The scale bar represents branch length. Reliability of internal branches was assessed using the bootstrap method with 1,000 pseudo-replicates. Bootstrap values greater than 70% are indicated at the nodes. Scale bar represents the number of substitutions per site. An unrooted tree was generated using NJplot.

The chromosomes of *L. garvieae* ATCC 49156 and Lg2 are 1,950,135 base pairs (bp) and 1,963,964 bp in size and contain 1,947 and 1,968 predicted protein-coding genes. Neither strain carries a plasmid. The general features of both genomes are summarized in Table 1 and Figure 1. An alignment analysis revealed their co-linearity (Fig. 2A) and 99% sequence identity in the aligned regions (1,942,687 bp). The strains share 1,944 orthologous genes. The 24 Lg2-specific genes that were absent in the ATCC 49156 genome included a 16.5-kb capsule gene cluster (LCGL_0431-LCGL_0448) (Table 2). This finding is consistent with the transmission electron microscopic evidence showing that Lg2 is encapsulated and ATCC 49156 is non-encapsulated [8]. Both genomes carried three types of IS element (IS982, IS-LL6, and ISSag5). IS982 were present in 7 and 11 copies in ATCC 49156 and Lg2, respectively. IS982 have been identified in the sequenced *L. lactis* genomes; *L. lactis* subsp. *cremoris* SK11 carries 55 copies [13]. Twelve pairs of adjacent IS-LL6 and ISSag5 elements, members of the IS3 family, were present in both genomes. Both genomes contained three prophage regions, two of which were short remnants (6 and 13 kb in size) with integrase genes; the other region (35 kb) contained 56 genes (LCGT_1094-LCGT_1149 in ATCC 49156 and LCGL_1114-LCGL_1169 in Lg2).

**Table 1:** General features of *L. garvieae* ATCC 49156 and Lg2.

	ATCC 49156	Lg2
Date of isolation	1974	2002
Country	Japan	Japan
Source	yellowtail	yellowtail
Virulence	avirulent	virulent
Serotype	KG^+^	KG^−^
Genome size (bp)	1,950,135	1,963,964
GC content (%)	38.8	38.8
Protein-coding genes	1,947	1,968
assigned function	1,120	1,137
conserved hypothetical	556	559
unknown function	205	206
phage-related	66	66
tRNA genes	62	62
rRNA operons	5	5

**Table 2:** Genes present in *L. garvieae* Lg2 but not in *L. garvieae* ATCC 49156.

Locus	Predicted gene product
LCGL_0431	transposase
LCGL_0432	transcription regulator
LCGL_0433	conserved hypothetical protein
LCGL_0434	chain length regulator
LCGL_0435	kinase
LCGL_0436	phosphatase
LCGL_0437	sugar transferase
LCGL_0438	rhamnosyltransferase
LCGL_0439	glycosyltransferase
LCGL_0440	acetyltransferase
LCGL_0441	glycosyltransferase
LCGL_0442	oligosaccharide repeat unit polymerase
LCGL_0443	flippase
LCGL_0444	UDP-glucose 6-dehydrogenase
LCGL_0445	conserved hypothetical protein
LCGL_0446	conserved hypothetical protein
LCGL_0447	truncated transposase
LCGL_0448	transposase
LCGL_0506	transposase
LCGL_0658	truncated transcriptional antiterminator
LCGL_0758	transposase
LCGL_1091	truncated preprotein translocase SecA
LCGL_1233	transposase
LCGL_1471	truncated cytochrome D ubiquinol oxidase subunit I

The genus *Lactococcus* is included within the family *Streptococcaceae*. We constructed a phylogenetic tree for concatenated sequences of ribosomal proteins from sequenced *Streptococcaceae*. *L. garvieae* and *L. lactis* were genealogically distinct (Fig. 2B). Using bidirectional best-hit analysis of all protein sequences, the genome of Lg2 was compared with those of *L. lactis* subsp. *lactis* IL1403 [12], *L. lactis* subsp. *lactis* KF147 [15], *L. lactis* subsp. *cremoris* MG1363 [11], *L. lactis* subsp. *cremoris* NZ9000 [14], and *L. lactis* subsp. *cremoris* SK11 [13]. The *L. garvieae* genome (1.9 Mb) was smaller than the five sequenced *L. lactis* genomes (2.3–2.6 Mb) (Table S1). Whole-genome alignment of the *L. garvieae* genome with the *L. lactis* genomes revealed a broken X-pattern (Fig. 2A), suggesting that the *L. garvieae* and *L. lactis* genomes have undergone multiple chromosomal inversions after their divergence from a common ancestor.

**Figure 3:**
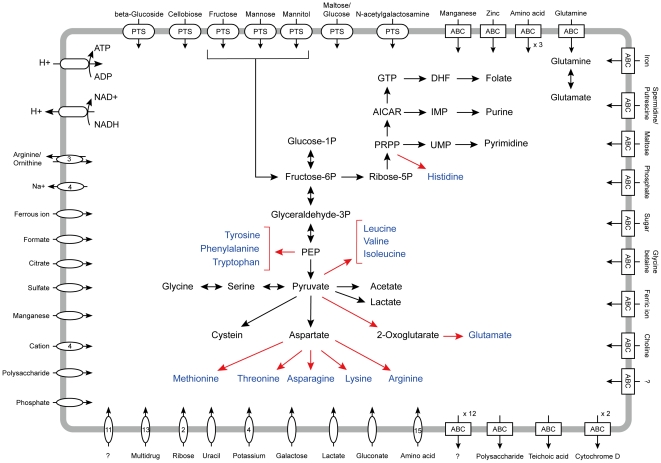
Predicted metabolic pathways in L. garvieae ATCC 49156. Red arrows indicate pathways that are not identified in *L. garvieae* but identified in *L. lactis* subsp. *lactis* IL1403.

Of all the predicted protein-coding genes in the Lg2 genome, 1,211 orthologs were common to the five *L. lactis* genomes, and may constitute the core genome of lactococci, likely inherited from the common ancestor. Compared to the five *L. lactis* genomes, 484 genes (25%) were specific to Lg2 and were dominated by hypothetical proteins or proteins of unknown function, which may include functions to cause disease in fish or to survive in the environment (Table S2). Thus, 75% of the protein-coding genes of Lg2 were orthologous to protein-coding genes of *L. lactis*, suggesting that *L. garvieae* and *L. lactis* still share a substantial portion of their overall physiology and metabolism. Conversely, the five *L. lactis* strains shared 1,579 protein-coding genes, of which 366 (23%) orthologs were absent in Lg2 (Table S3). The 366 genes contained many genes involved in amino acid biosynthesis. *L. garvieae* retained fewer genes for amino acid transport and metabolism (category E) than *L. lactis* using the clusters of orthologous groups (COGs) database (Fig. S1). The capacity for amino acid biosynthesis by *L. garvieae* was limited to aspartate, serine, glycine, and cysteine (Fig. 3). The incompleteness of amino acid biosynthesis in *L. garvieae* is a typical functional feature of most pathogens with small genome size like clostridia [22]. On the other hand, the *L. garvieae* genomes encoded 3 ATP-binding cassette (ABC) transporters and 15 permeases for amino acids or peptides (Fig. 3), some of which are included in *L. garvieae* (Lg2)-specific genes listed in Table S2. These transporters are complimented by >10 peptidases and proteases, and may be involved in the uptake via the destruction of host tissue.

### Capsule gene cluster of Lg2

**Figure 4:**
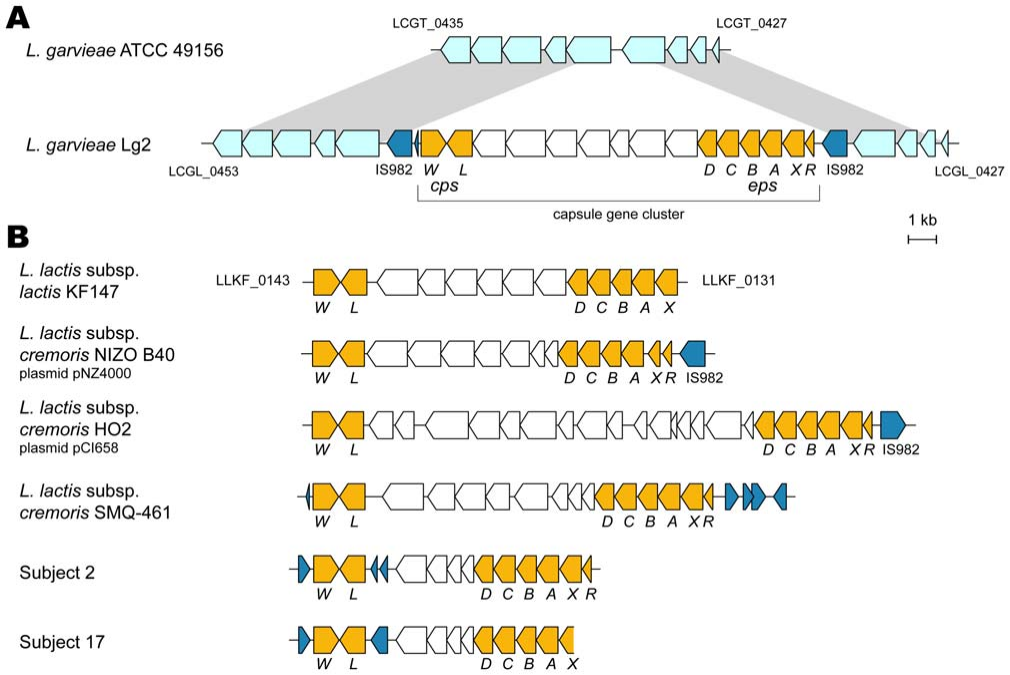
The capsule gene cluster arrangement in *L. garvieae* Lg2 and *L. lactis* strains. Genes and their orientations are depicted with arrows using the following colours: orange, conserved genes in the capsule gene cluster; blue, transposase genes; white, other genes in the capsule gene cluster. Gray bars indicate orthologous regions. (**A**) Comparisons of the genomic location of the capsule gene cluster in *L. garvieae* Lg2 and the corresponding region in *L. garvieae* ATCC 49156. (**B**) The capsule gene cluster arrangement in *L. lactis* strains and the human gut bacteria (Subjects 2 and 17). Genes and their orientations are depicted with arrows using the same colour coding as described in (A). Accession numbers of the sequences used in this figure are as follows: *L. lactis* subsp. *cremoris* NIZO B40, AF036485; *L. lactis* subsp. *cremoris* HO2, AF142639; *L. lactis* subsp. *cremoris* SMQ-461, AY741550; Subject 2, AB612914; Subject 17, AB612915.

Encapsulated strains of *L. garvieae* have been reported to be resistant to opsonophagocytosis and host serum killing [6], [7]. The 16.5-kb capsule gene cluster found in Lg2 was composed of 15 genes flanked on both ends by IS982 elements (LCGL_0431 and LCGL_0448) (Table 3 and Fig. 4A). The GC content (31%) of this 16.5-kb gene cluster differed from the chromosomal average (39%). Comparison of this genetic locus with the corresponding locus in the sequenced *L. lactis* genomes revealed that the gene cluster was apparently inserted into the locus syntenic to the sequenced lactococci (Fig. S2). These structural features clearly indicated that the capsule gene cluster of Lg2 is a genomic island. The corresponding capsule gene cluster in avirulent strain *L. garvieae* ATCC 49156 might have been lost during its subculturing, because ATCC 49156 was originally isolated from diseased yellowtail and could have been virulent [16].

**Table 3:** Properities and similarities of the capsule gene cluster in *L. garvieae* Lg2.

Locus	Gene	Best BLASTP hit	Accession No.	Amino acid identity (%)	Predicted gene product
LCGL_0431	IS982	*L. lactis* SK11	ABJ72537	87	transposase
LCGL_0432	*epsR*	*L. lactis* HO2	AAP32713	100	transcription regulator
LCGL_0433	*epsX*	*L. lactis* SMQ-461	AAX19699	98	conserved hypothetical protein
LCGL_0434	*epsA*	*L. lactis* SMQ-461	AAX19700	97	chain length regulator
LCGL_0435	*epsB*	*L. lactis* Ropy352	ABN47386	97	kinase
LCGL_0436	*epsC*	*L. lactis* HO2	AAP32717	99	phosphatase
LCGL_0437	*epsD*	*L. lactis* SMQ-461	AAX19703	93	sugar transferase
LCGL_0438		*S. mitis*	BAD22640	64	rhamnosyltransferase
LCGL_0439		*S. pneummoniae*	CAI33605	38	glycosyltransferase
LCGL_0440		*S. pneummoniae*	CAI33463	47	acetyltransferase
LCGL_0441		*S. pneummoniae*	CAI33772	38	glycosyltransferase
LCGL_0442		*S. thermophilus*	AAN63689	24	oligosaccharide repeat unit polymerase
LCGL_0443		*L. lactis* KF147	ABX75688	40	flippase
LCGL_0444		*C. spiroforme*	EDS75375	71	UDP-glucose 6-dehydrogenase
LCGL_0445	*cpsL*	*L. lactis* SMQ-461	AAX19712	95	conserved hypothetical protein
LCGL_0446	*cpsW*	*L. lactis* SMQ-461	AAX19713	98	conserved hypothetical protein
LCGL_0447		*L. lactis* MG1363	CAL97152	87	truncated transposase
LCGL_0448	IS982	*L. lactis* SK11	ABJ73328	98	transposase

Of the 15 genes, eight genes (*epsRXABCD* and *cpsLW*) were conserved in the exopolysaccharide (EPS) biosynthesis gene cluster in four *L. lactis* strains with amino acid identity of >93% to their orthologs as well as IS982 in two *L. lactis* strains (Table 3 and Fig. 4B) [23]–[26]. Also, the *L. lactis* KF147 genome had almost identical gene organization to that in Lg2 (Fig. 4B) at a different locus on the chromosome (Fig. S2). Again, these data indicate that the capsule gene cluster may have been wildly spread among lactococci as genomic islands. The predicted genes located between *cpsL* and *epsD* in the five strains were much less conserved and varied in number among them. The seven genes (LCGL_0438-LCGL_0444) between *cpsL* and *epsD* in Lg2 had a low but significant sequence similarity (24–71%) with genes mostly in *Streptococcus* spp. (Table 3). The seven genes appeared to additionally encode enzymes involved in architecture of polysaccharides of capsule [27].

*L. garvieae* Lg2-S, which was spontaneously generated during subculturing of Lg2, is less virulent than Lg2, exhibits a KG+ phenotype, and is non-encapsulated [8]. The sequence analysis of the capsule gene cluster of Lg2-S identified a single base deletion in the *cpsL* (conserved hypothetical protein) and *epsD* (glycosyltransferase) genes, respectively. RT-PCR revealed that mRNAs of all 15 genes in the capsule gene cluster were expressed in Lg2-S at the almost same level as in capsulated Lg2 (Fig. S3). The *cpsL* and *epsD* genes were highly conserved among *L. lactis* strains (Fig. 4), suggesting the importance of these two genes in EPS synthesis. Glycosyltransferase encoded by *epsD* mediates the first step of EPS biosynthesis [22], [24] and the frameshift in *epsD* of Lg2-S resulted in the loss of C-terminal functional domain (Pfam PF02397). Thus, mutations in *cpsL* and *epsD* may be responsible for the non-encapsulated phenotype of less virulent Lg2-S.

### Potential virulence factors in *L. garvieae*

Aside from the capsule gene cluster, we identified possible virulence genes in the Lg2 genome by similarity search to those annotated or validated in other pathogenic bacterial genomes. It has been known that Lg2 is α-haemolytic, due to the express of bacterial haemolysins [8]. The analysis showed that the Lg2 genome contained three candidate genes encoding haemolysins. The LCGL_0323 protein shared 56% amino acid identity with haemolysin (accession number AAO81463) in *Enterococcus faecalis*, and contained the motif (PF03006) conserved in proteins with haemolytic activity. The LCGL_0597 protein shared 72% amino acid identity with haemolysin (accession number EEF64743) in *Streptococcus suis*. The LCGL_0374 protein showed 59% amino acid identity to haemolysin (accession number AAK33420) in *Streptococcus pyogenes* and contained a cleavable N-terminal signal sequence.

NADH oxidase and superoxide dismutase (SOD) are also potential virulence factors [28]. Both enzymes serve for survival of pathogens in aerobic environments by conferring them to tolerance to oxygen and reactive superoxide radicals. The LCGL_0664 protein shared 51% amino acid identity with NAD oxidase (SP1469) in Streptococcus pneumoniae TIGR4, and the LCGL_0285 protein shared 68% amino acid identity with SOD (SP0766) in *S. pneumoniae* TIGR4. The LCGL_1596 protein shared 68% amino acid identity with phosphoglucomutase (accession number AAW56093), a virulence factor conferring the resistance to peptide antimicrobials in a fish pathogen *Streptococcus iniae* [29].

Adhesion to host tissues represents a first crucial step in most bacterial infections. The Lg2 genome possessed three genes (PavA, enolase, and PsaA) having significant sequence similarity to virulence determinants exposed on the cell surface of pathogenic streptococci. PavA and enolase are pneumococcal surface adhesins binding to extracellular matrix component [28], [30]. The LCGL_1330 protein shared 62% amino acid identity with Pav (SP0966) in S. pneumoniae TIGR4, and contained the fibronectin-binding motif (PF05833). The LCGL_1514 protein shared 89% amino acid identity with enolase (SP1128) in *S. pneumoniae* TIGR4. PsaA is a divalent metal-ion-binding lipoprotein component of an ABC transport system [28], [30]. The LCGL_1533 protein shared 49% amino acid identity with PsaA (SP1650) in *S. pneumoniae* TIGR4.

Sortase likely plays a universal role in the virulence of Gram-positive bacteria, and is responsible for anchoring all LPxTG-containing surface proteins [31]. The Lg2 genome contained four genes (LCGL_1005, LCGL_1410, LCGL_1585, and LCGL_1672) encoding proteins with an LPxTG-type motif for covalent anchoring to the peptidoglycan matrix, and these proteins can be cleaved by sortase (LCGL_1312). Of the four genes, the LCGL_1585 protein contained the collagen-binding domain (PF05737), and the LCGL_1005 and LCGL_1672 proteins contained the mucin-binding domain (PF06458). The LCGL_0196 protein had LPIVG motif at the C-terminus and contained a domain (PF05738) conserved in the collagen-binding surface protein of *Staphylococcus aureus*.

Lg2 had a cluster (LCGL_0842-LCGL_0845) encoding proteins with a C-terminal WxL domain, which is conserved in the surface proteins of low-GC Gram-positive bacteria and attaches to peptidoglycan on the cell surface [32]. The proteins (LCGL_0842 and LCGL_0845) having WxL domain were present together with the protein (LCGL_0843) having DUF916 domain (PF06030) of unknown function and the small protein (LCGL_0844) with LPxTG-like sorting motif. This gene organization is similar to that in *Lactobacillus plantarum* [33].

### Detection of the capsule gene cluster in healthy human faecal bacteria

Using the consensus sequence of the capsule gene cluster between Lg2 and KF147, consensus PCR primers were designed to examine the presence of the capsule gene cluster in human faecal bacterial DNA. PCR was done for the bacterial DNAs isolated from several human faecal samples, and the PCR products obtained from two samples (Subjects 2 and 17) were sequenced. The sequence analysis revealed the presence of orthologs in the capsule gene cluster in these two samples, showing that the capsule gene cluster might be prevalent among lactococci.

The eight genes (*epsRXABCD* and *cpsWL*) in Subject 2 and the seven genes (*epsXABCD* and *cpsWL*) in Subject 17 were highly conserved among the capsule gene clusters in lactococci (Fig. 4B), showing high similarity (93–100% amino acid sequence identity) to those of *L. lactis* and *S. thermophilus*, which have been involved in fermenting dairy products as GRAS (Table S5). The genes except transposase genes located between *cpsL* and *epsD* in Subjects 2 and 17 also showed high similarity (98–100% amino acid sequence identity) to those of *L. lactis* and *S. thermophilus* (Table S5), indicating that orthologs in the capsule gene cluster found in the human microbiomes might come from non-pathogenic bacteria in the human gut. Thus, products encoded by these orthologs in the human microbiomes may cause no damage to the human host.

### Conclusion

The complete genome sequences of *L. garvieae* ATCC 49156 and Lg2 were determined. A comparative genome analysis indicated that virulent strain Lg2 contains a specific capsule gene cluster that is not found in a virulent strain ATCC 49156. Furthermore, the less virulent strain Lg2-S contained two disrupted genes in the capsule gene cluster, showing the correlation of the gene disruption and phenotype of Lg2-S. These results, combined with the previous report using transmission electron microscopy [8], clearly indicate that the capsule is crucial for full virulence of *L. garvieae*. The present data also showed that the capsule gene cluster is a genomic island and may have been widely spread among lactococci including those in human gut microbiota. In addition to the capsule gene cluster, a number of other genes possibly involved in virulence factors were identified in Lg2, which remained to be elucidated for pathogenesis in fishes.

## Supporting Information

Figure S1 **Comparative analysis by functional categories of the gene repertoires of** ***L. garvieae*** **and** ***L. lactis*** **.** The number of genes on each genome within each functional category as defined by the COG database is shown.
(TIF)

Figure S2 **Comparisons of the genomic location of the capsule gene cluster in** ***L. garvieae*** **Lg2 and the corresponding location of** ***L. garvieae*** **ATCC 49156 and** ***L. lactis*** **strains.** Genes and their orientations are depicted with arrows. Red indicates the capsule gene cluster including IS982 elements. Gray bars indicate orthologous regions.
(TIF)

Figure S3 **mRNA expression analysis of the genes in the capsule gene cluster of** ***L. garvieae*** **Lg2 and Lg2-S.** Total RNA from *L. garvieae* Lg2 and Lg2-S was extracted and was subjected to semi-quantitative RT-PCR. The PCR products were electrophoresed, stained, and photographed.
(TIF)

Table S1 **General genomic features of** ***L. garvieae*** **and five other lactococci.**
(XLSX)

Table S2 **The 484 genes of** ***L. garvieae*** **Lg2 that are absent in any of the five sequenced** ***L. lactis*** **.**
(XLSX)

Table S3 **The 366 genes conserved in the five sequenced** ***L. lactis*** **but absent in** ***L. garvieae*** **Lg2.**
(XLSX)

Table S4 **Primer sequences used for RT-PCR.**
(XLSX)

Table S5 **Properities and similarities of the capsule gene clusters in the human gut microbiome (Subjects 2 and 17).**
(XLSX)
