# Supplementary figures and images for: Complete Genome Sequence and Comparative Analysis of the Fish Pathogen Lactococcus garvieae
Source: PLoS One. 2011 Aug 4;6(8):e23184. doi: 10.1371/journal.pone.0023184 (PMC3150408; doi:10.1371/journal.pone.0023184)

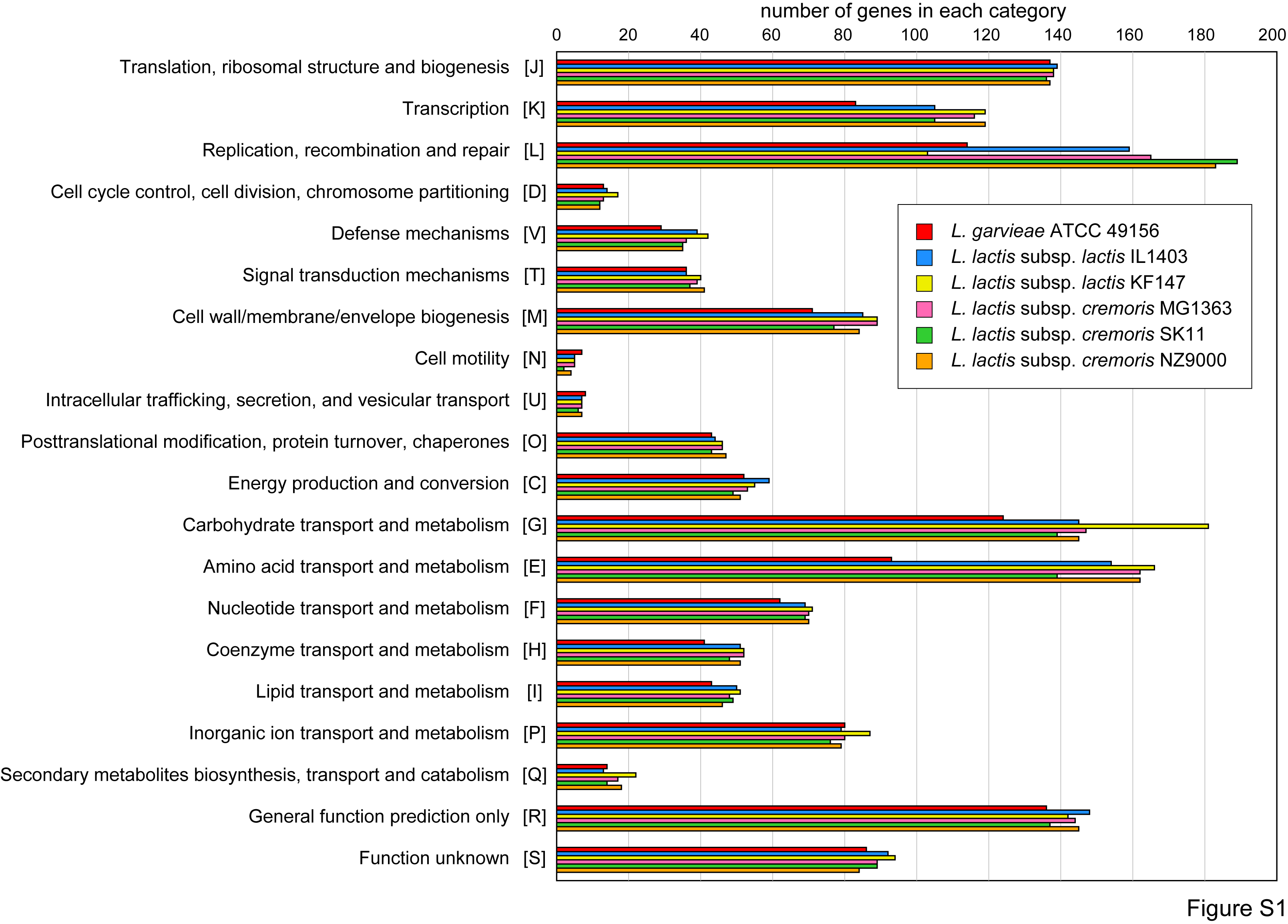

Supplement: Figure S1 — Comparative analysis by functional categories of the gene repertoires of L. garvieae and L. lactis . The number of genes on each genome within each functional category as defined by the COG database is shown. (TIF) [file pone.0023184.s001.tif]

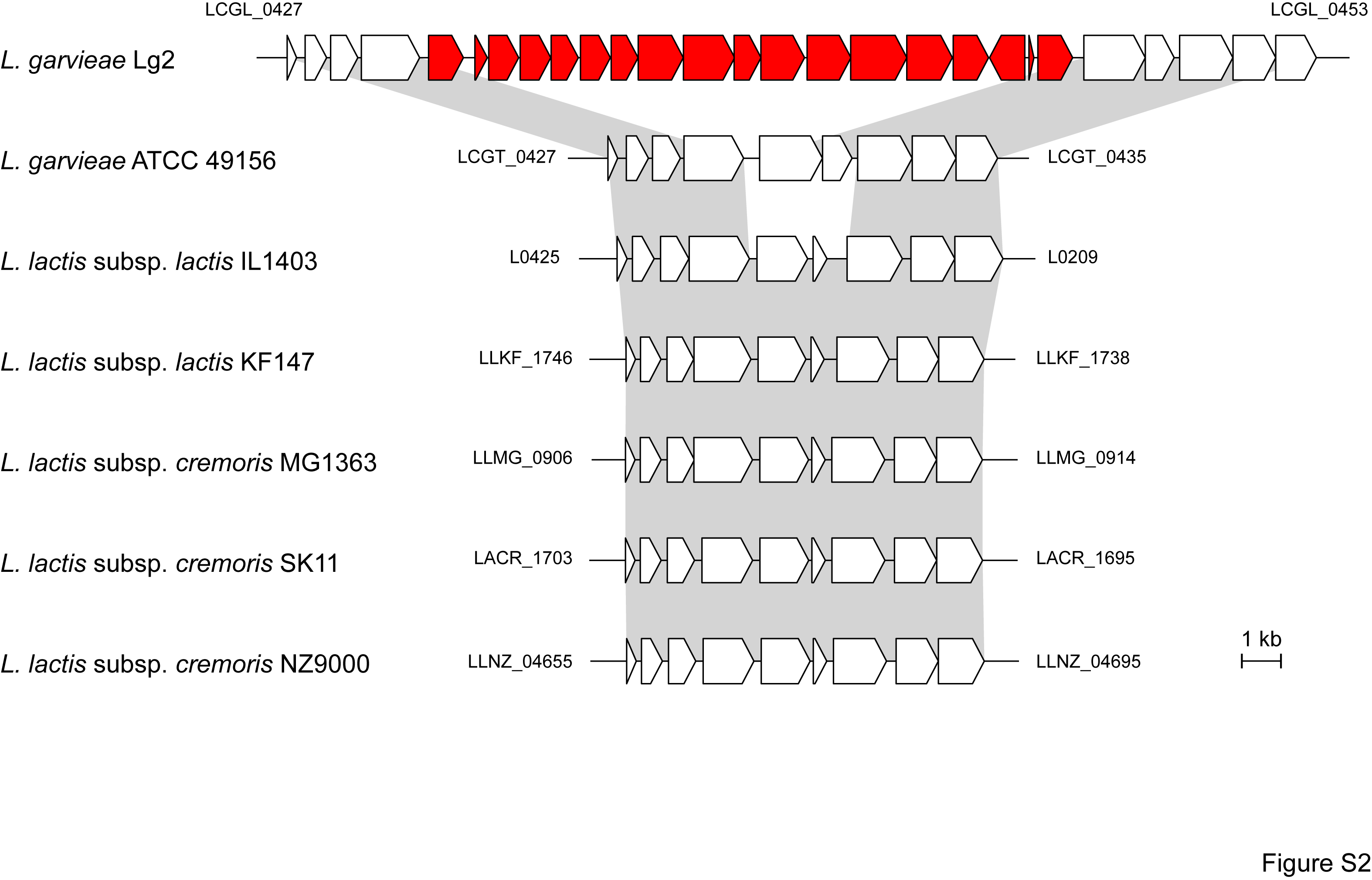

Supplement: Figure S2 — Comparisons of the genomic location of the capsule gene cluster in L. garvieae Lg2 and the corresponding location of L. garvieae ATCC 49156 and L. lactis strains. Genes and their orientations are depicted with arrows. Red indicates the capsule gene cluster including IS982 elements. Gray bars indicate orthologous regions. (TIF) [file pone.0023184.s002.tif]

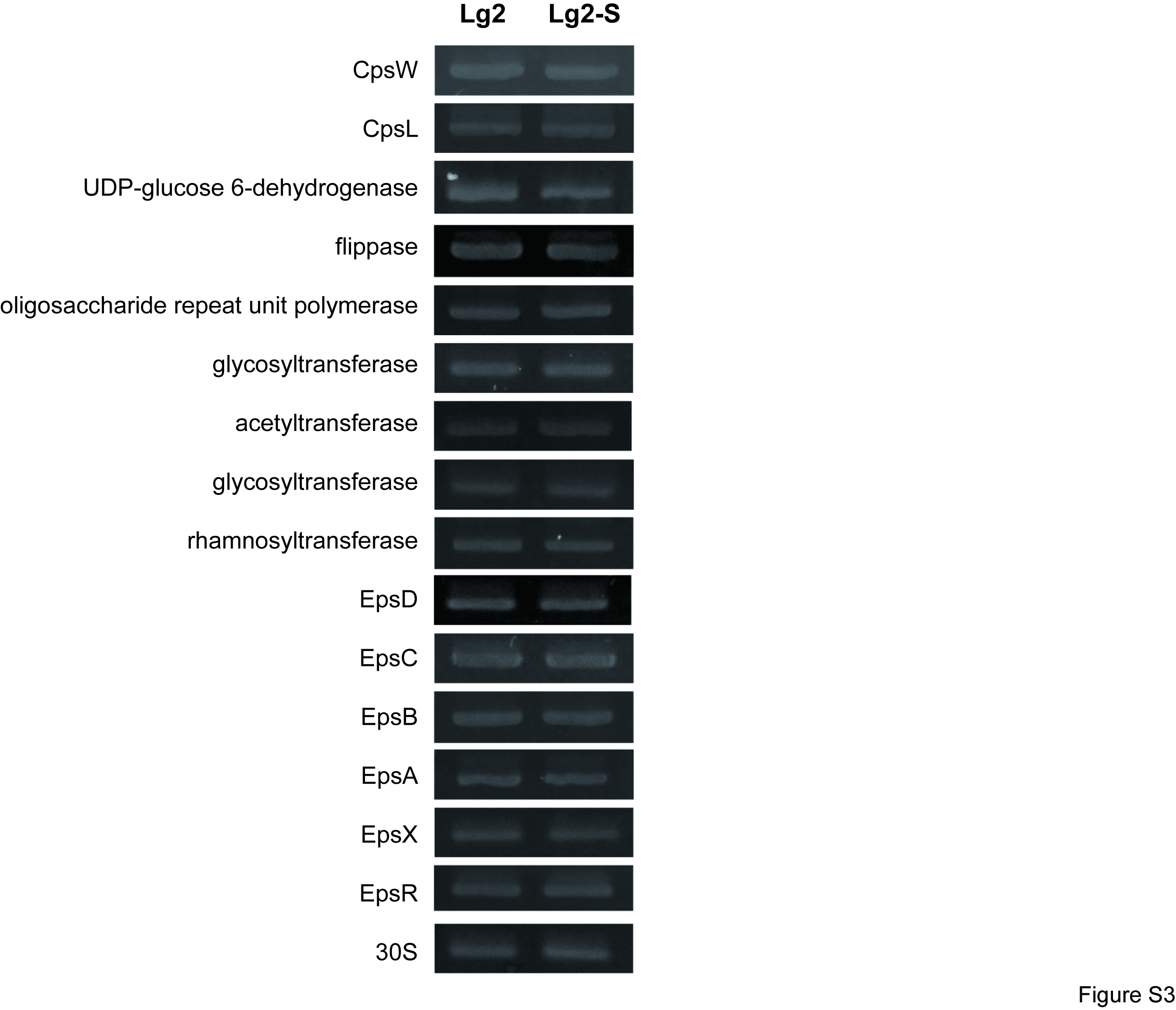

Supplement: Figure S3 — mRNA expression analysis of the genes in the capsule gene cluster of L. garvieae Lg2 and Lg2-S. Total RNA from L. garvieae Lg2 and Lg2-S was extracted and was subjected to semi-quantitative RT-PCR. The PCR products were electrophoresed, stained, and photographed. (TIF) [file pone.0023184.s003.tif]
